# Distraction osteogenesis in a severe mandibular deficiency

**DOI:** 10.1186/1746-160X-3-7

**Published:** 2007-01-20

**Authors:** Kerim Ortakoglu, Seniz Karacay, Metin Sencimen, Erol Akin, Aykut H Ozyigit, Osman Bengi

**Affiliations:** 1 Department of Oral and Maxillofacial Surgery, Gulhane Military Medical Academy, Ankara, Turkiye; 2 Department of Orthodontics, Gulhane Military Medical Academy, Ankara, Turkiye

## Abstract

**Objective:**

Distraction osteogenesis is an alternative treatment method for the correction of mandibular hypoplasia. In this case report, distraction with a multidirectional extraoral device was performed to gradually lengthen the corpus and ramus of a patient who had a severe hypoplastic mandible.

**Materials and methods:**

The patient underwent bilateral extraoral ramus and corpus distraction osteogenesis. After seven days of latency period, distraction was performed 0.5 mm twice a day. Subsequent consolidation period was 12 weeks.

**Results:**

The patient's mandible was elongated successfully. Cephalometric analysis revealed that ANB angle decreased from 13° to 6°, overjet of 15 mm decreased to 4 mm, corpus length increased from 49 mm to 67 mm, and ramus length increased from 41 mm to 43 mm. Posterior airway space (PAS) also increased due to advancement of the mandible. In stereolithographic model evaluation it was determined that the distances from condylion to gonion and from gonion to pogonion increased.

**Conclusion:**

Satisfactory results from both aesthetic and functional standpoints were obtained by distraction osteogenesis of the ramus and corpus.

## Background

Distraction osteogenesis (DO) is a biologic process of new bone formation between the surfaces of bone segments that are gradually separated by incremental traction [1]. A callus forms between the separated bone segments and as long as the traction proceeds, callus tissues are stretched inducing the new bone formation [2].

DO was first introduced by Codivilla at the beginning of twentieth century and during 1950s, the studies of Ilizarov made a contribution in the development of the technique by elucidating the biological and mechanical principals in the formation of new bone [3-5]. DO has been applied to craniofacial region since McCarthy et al [6] reported the first clinical application of the technique in the treatment of four children with either unilateral or bilateral mandibular hypoplasia.

In the lengthening of the hypoplastic mandible, external or intraoral devices have been used. Although intraoral distractors are more convenient socially and leave no residual skin scars, extraoral bidirectional or multidirectional devices should be preferred in severe hypoplastic cases, when three-dimensional vector control is required and gonial angle control is necessary [1].

In this case report, we intended to present the treatment of an adult patient who had severe mandibular deficiency. An extraoral multidirectional distractor was used to achieve independent horizontal distraction of the corpus and vertical distraction of the ramus. Amount of lengthening was determined with cephalograms and stereolithographic models. Stereolithographic modelling is a well known system Moreover alterations at the posterior airway space due to elongation of mandible were also presented.

## Case report

### Diagnosis

A 23-year old male patient who had severe mandibular hypoplasia referred to the Department of Oral and Maxillofacial Surgery for treatment. His main complaints were unaesthetic appearance, snoring, wheezing, and difficulties during respiration, speech, and chewing.

**Figure 1:**
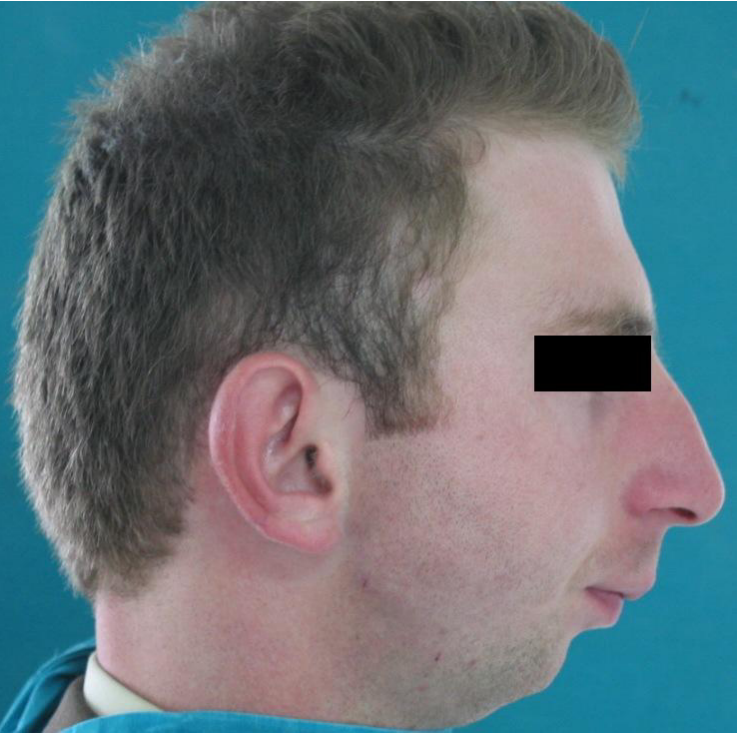
Extraoral lateral view of the patient before treatment.

**Figure 2:**
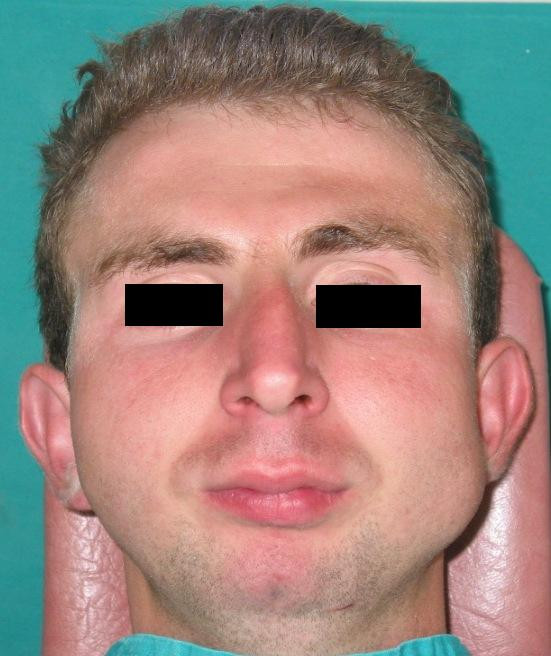
Extraoral front view of the patient before treatment.

**Figure 3:**
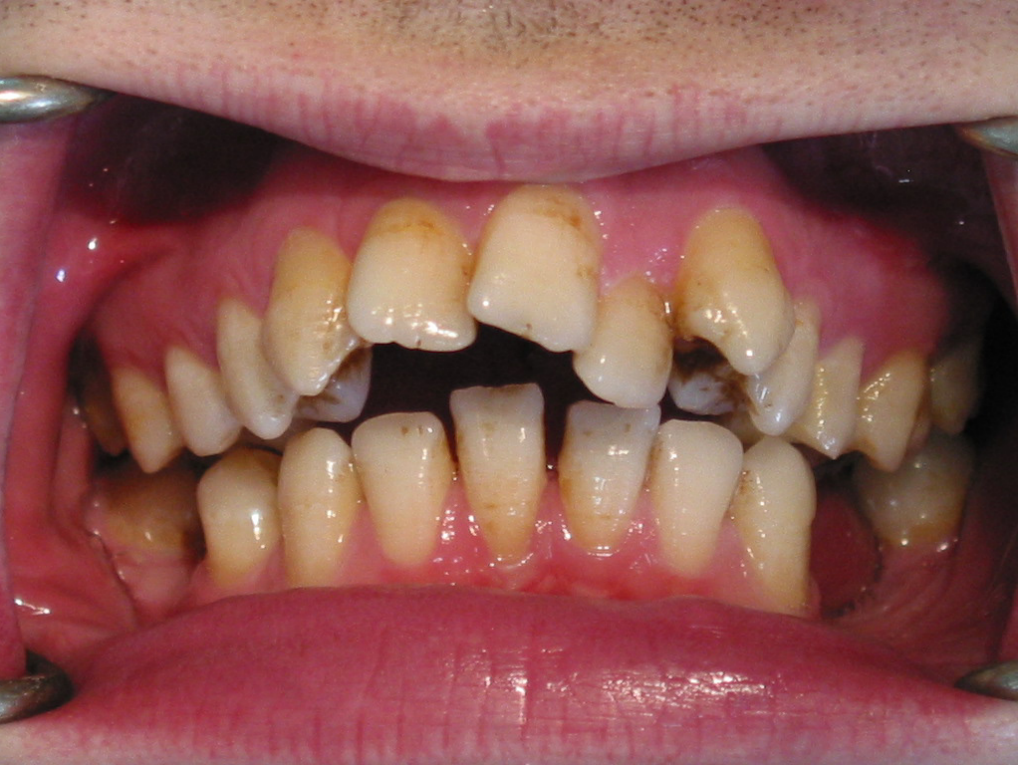
Intraoral view of the patient before treatment.

In the extraoral examination, severely convex profile with a receding chin and a prominent nose was observed. Intraoral examination revealed a Class II Division I malocclusion with an excessive overjet. Maxillary arch had a triangular form. Right lateral incisor was missing, mild rotation of the left central incisor, palatoversion of the left lateral incisor and buccoversion of the left canine were observed due to arch length deficiency. Lower second premolars, first and second molars were missing at both sides and first premolar was also absent at left side. Mesial tipping of right and left third molars was observed due to missing of the adjacent teeth (Figure 1, 2, and 3).

To determine the skeletal deformity, cephalometric analysis and measurements on stereolithographic three dimensional skull prototype model obtained through three dimensional computerized tomographic images of the patient were used. The stereolithographic model was built by Spectrum Z510 Color 3D Printers (Z Corporation-Burlington, USA).

**Figure 4:**
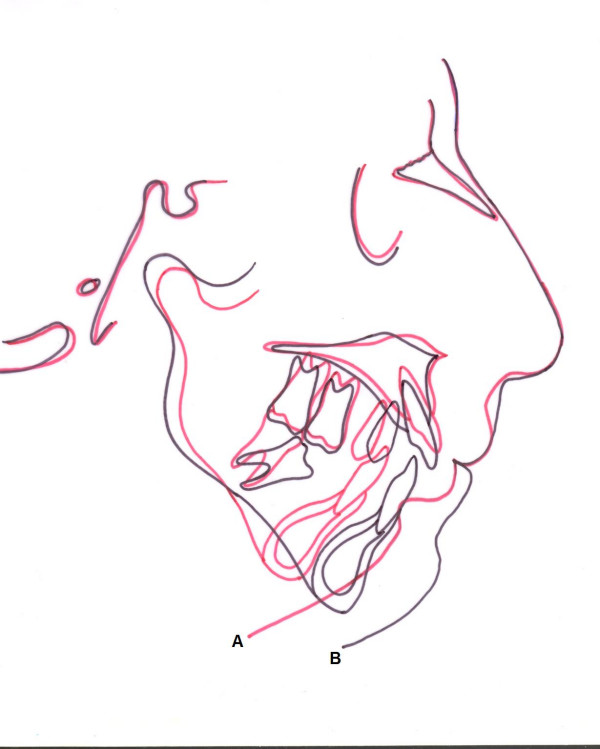
Cephalometric superimposition on total face before (A) and after treatment (B).

**Figure 5:**
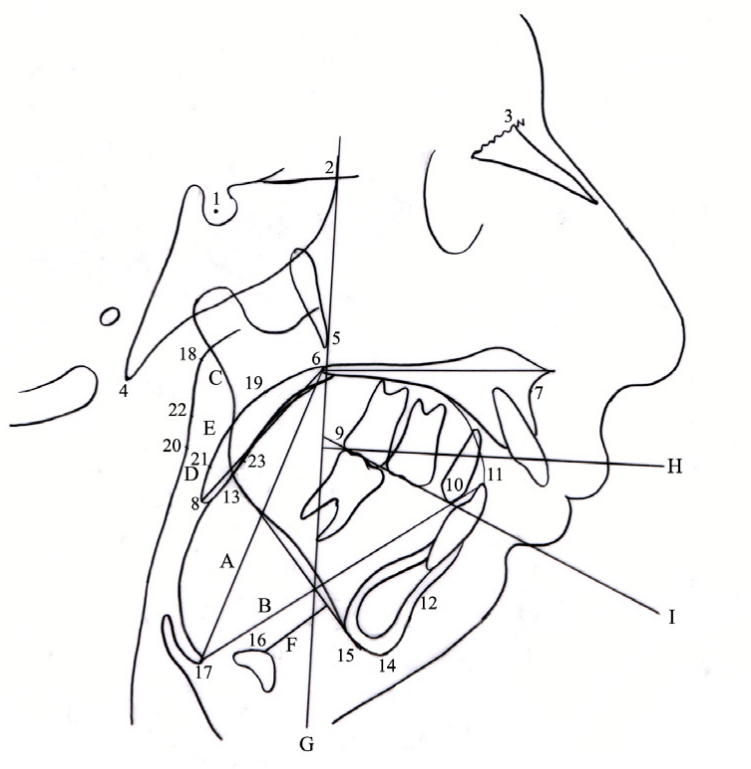
Cephalometric landmarks (numeric) and planes (capital letter) used for posterior airway evaluation. 1) S: sella turcica; 2) Se: sphenoethmoidal junction; 3) N: nasion; 4) Ba: basion; 5) Ptm: pterygomaxillary fissure; 6) PNS: posterior nasal spine; 7) A: A-point, subspinale; 8) P: ate, the most inferior tip of the soft palate; 9) Mo: posterior contact point of the molar; 10) PreM: contact point of the premolar;11) Tt: tip of the tongue; 12) B: B-point, supramentale;13) Go: gonion; 14) Gn: gnathion; 15) Me: menton; 16) H: hyoidale; 17) Eb: base of the epiglottis; 18) PhwS: superior posterior pharyngeal wall; 19) Psp: posterior-superior palate, the most posterior-superior point of the soft palate; 20) PhwN: posterior pharyngeal wall at narrowest point; 21) STBn: most anterior point in the airway on the soft palate; 22) PhwI: inferior posterior pharyngeal wall; 23) STBi: most anterior point in the airway on the tongue; A) VAL = PNS to Eb; B) TL = Eb to Tt; C) S-PAS = PhwS to Psp; D) N-PAS = PhwN to STBn; E) I-PAS = PhwI to STBi; F) H-MP: H to mandibular plane; G) PM: extends down from SE point through the inferior point of PTM; H) NOA: neutral occlusal axis, perpendicular to PM through the posterior-inferior-most contact point of the last fully erupted maxillary molar; I) FOP: functional occlusal plane.

Cephalometric analysis indicated severe skeletal Class II discrepancy confirmed by an ANB of 13°. Point A and point B were 6 mm and 42 mm behind the nasion vertical (NV), respectively. These norms supported each other and revealed that the patient had a severely retruded mandible. Ramus length (Ar-Go) was 41 mm and corpus length (Go-Gn) was 49 mm revealing deficiency of both ramus and corpus. Increases in the mandibular plane, gonial angle and Y-axis emphasized the posterior rotation of the mandible. The anterior facial height was 129 mm, posterior facial height was 64 mm and the ratio of these values was 49.61 % revealing that the patient had a long face. Overjet and overbite were measured as 15 mm and 4 mm, respectively. The cephalometric landmarks used in our report are demonstrated in Figures 4, and 5 and the pre and post treatment measurements of the cephalograms are presented in Table 1.

**Table 1:** Cephalometric analysis of the patient

	**Pre-treatment**	**Post-treatment**
**SNA**	76°	76°
**SNB**	63°	70°
**ANB**	13°	6°
**NV-A**	6 mm	6 mm
**NV-Pog**	-42 mm	-24 mm
**S-N**	73	73
**S**	123°	120°
**Ar**	142°	153°
**Go**	170°	145°
**Ar-Go**	41 mm	43 mm
**Go-Gn**	49 mm	67 mm
**Y Axis**	78°	69°
**SN/ANS-PNS**	16°	16°
**SN/Occ.**	26°	12°
**SN/Go-Gn**	73°	59°
**ANS-PNS/Go-Gn**	58°	44°
**Co-A**	82 mm	82 mm
**Co-Pog**	95 mm	114 mm
**N-Me**	129 mm	134 mm
**N-ANS**	59 mm	59 mm
**ANS-Me**	80 mm	77 mm
**S-Go**	64 mm	67.5 mm
**S-Go/N-Me**	49.61%	50.37%
**1/SN**	101°	96°
**1/Go-Gn**	65°	68°
**1/1**	120°	137°
**1/NA**	25°	21°
**1-NA**	4 mm	5 mm
**1/NB**	22°	17°
**1-NB**	9 mm	7 mm
**E-Line**	-3 mm/-2 mm	-4 mm/-3 mm
**Overjet**	15 mm	4 mm
**Overbite**	4 mm	1 mm

Ramus and corpus lengths were also measured on the stereolithographic models. The preoperative distance between condyle and gonion was 50 mm and the distance between gonion and menton was 69 mm for the right side. They were 44 mm and 68 mm, respectively for the left side. The patient had severe mandibular hypoplasia due to inadequate ramus and corpus length, therefore using an extraoral multiplanar distractor was inevitable. Tritrac^® ^External Distractor (Ucmed Medical Ltd. Co.-Ankara, Turkey) was used for the treatment of the presented case.

### Orthodontic treatment, surgical technique and distraction procedure

Preadjusted appliances (0.018 × 0.022 inch) were placed in the maxillary and mandibular arch and open coil spring was used to open space for the left lateral incisor. Following the leveling phase, 0.016 × 0.022 inch arch wires were placed on the upper and lower teeth.

Because of the limitation of the mouth opening, nasotracheal intubation was performed with fiberoptic bronchoscope. Under general anaesthesia, after completing the intraoral incisions and exposing the cortex of mandibular corpus and ramus on both sides, the device was secured to the mandible via 6 percutaneous pins. This system had 3 pairs of pins to be secured; one pair to the ramus, the other to the angulus and the last one to the corpus region inferior to the mandibular canal. During this procedure extraoral incisions and trochar were used. The mandibular osteotomy was then performed with round and fissure burs and osteotomes. Two osteotomies on each ipsilateral side were planned. One of the osteotomies was on the ramus and the other one was anterior to the second molar. After the completion of the osteotomies, the distractors were tested and were placed paralell to each other. Then, the incisions were primarily closed.

A 7 days of latency phase was waited for callus formation. Afterwards, distraction was performed at a rate of 0.5 mm, twice a day. Ramus was distracted for 7 days and corpus was distracted for 45 days. While the corpus was being distracted, the angles of distractors were reduced 5° on days 10, 20, 30, 40 so as to decrease the gonial angle. Paralellization of the distractors were protected during this procedure. Once the desired bone length had been acquired, 3 months of consolidation was allowed. Following the consolidation phase the distractor was removed under local anaesthesia and the extraoral wounds were debrided and primarily closed and left for recovery.

The patient used class II intraoral elastics at night time in order to prevent relapse and elastic traction was completed after 3 months. The patient was followed-up for one more month in order to determine whether relapse would occur. Brackets were debonded 4 months after consolidation period. The total treatment time was 14 months.

Finally the prosthetic rehabilitations of the missing teeth in the mandible were solved with stationary restorations.

## Results

**Figure 6:**
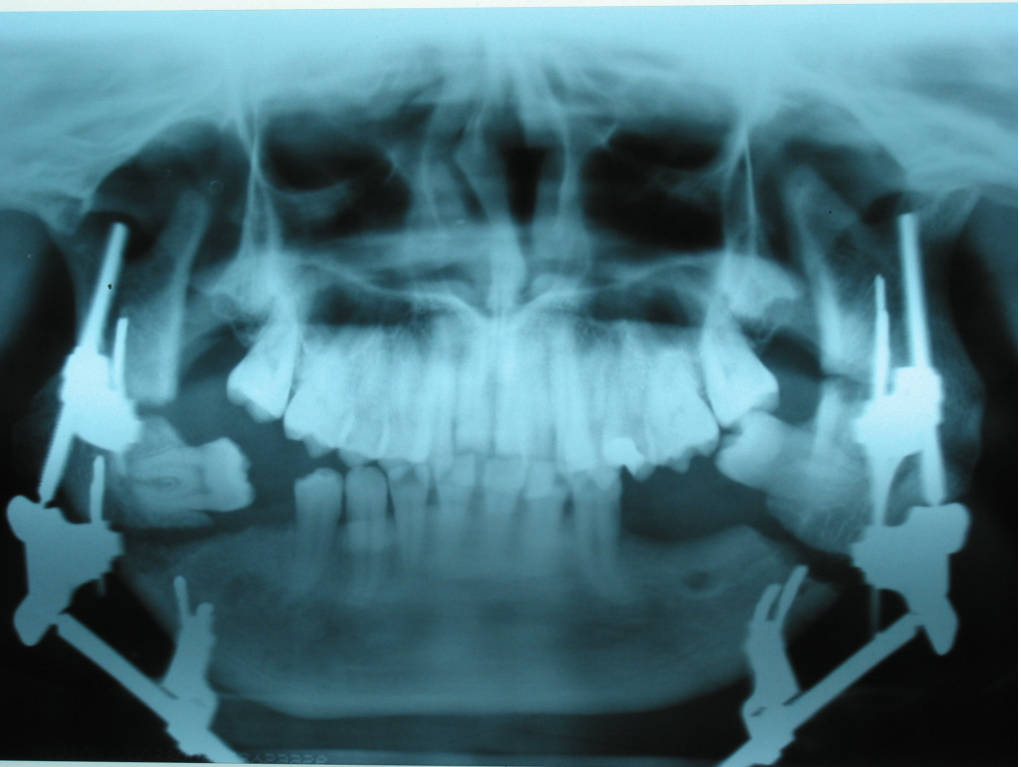
Panoramic radiograph taken at the beginning of distraction showing the osteomies.

**Figure 7:**
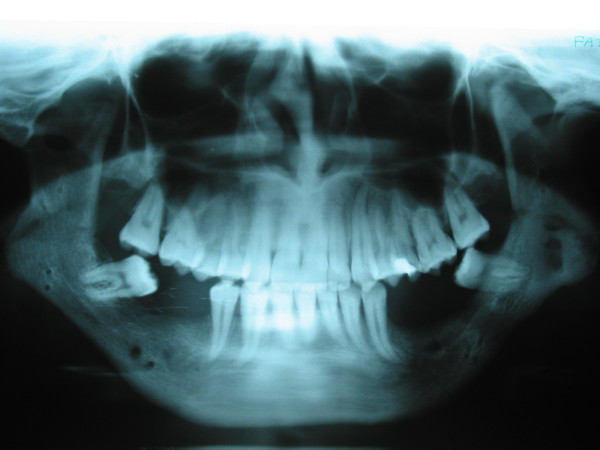
Radiograph taken after consolidation phase showing the formation of new bone at the distraction site.

No significant complications associated with the surgical procedure were encountered postoperatively. Panoramic radiographs were taken postoperatively to monitor the new bone formation. After the consolidation phase, the gaps were all filled with new bone (Figures 6, and 7).

Cephalometric analysis revealed that overjet of 15 mm and overbite of 4 mm decreased to 4 mm and 1 mm, respectively. ANB angle decreased from 13° to 6°, distance between pogonion and NV increased from 42 mm to 24 mm, corpus length (Go-Gn) increased from 49 mm to 67 mm, and ramus length (Ar-Go) increased from 41 mm to 43 mm showing the successful elongation of ramus and corpus. Gonial angle, inclination of the mandibular plane, and Y-axis decreased revealing the anterior rotation of the mandible. Elongation of the ramus length increased the posterior face height and anterior rotation of the mandible decreased the anterior face height. These alterations changed the profile of the patient and provided more symmetric and aesthetic appearance. Cephalometric superimpositions of total face on SN plane is demonstrated in Figure 4, and and pre-treatment and post-treatment cephalometric analysis are presented in Table 1.

**Figure 8:**
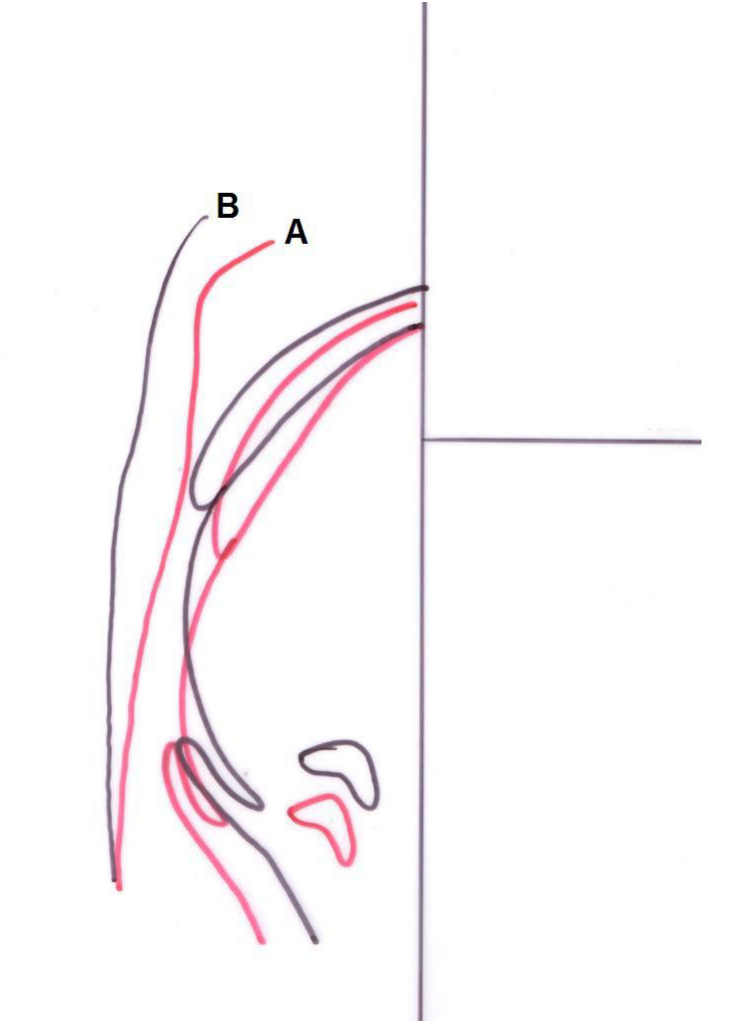
Cephalometric superimposition on PAS before (A) and after treatment (B).

Alterations in the posterior airway space (PAS) were also determined. The distance from Eb to posterior nasal spina increased from 72 mm to 75 mm while the distance from Eb to tongue tip decreased from 81 mm to 74 mm. All the PAS parameters increased after treatment but the most dramatic increase was observed at N-PAS distance as it increased from 7 mm to 20 mm. N-PAS that was under I-PAS before treatment, positioned between S-PAS and I-PAS following the treatment. The perpendicular distance from hyoid to mandibular plane decreased from 19 mm to 11 mm after treatment. Cephalometric landmarks used for posterior airway evaluation, soft and hard-tissue linear measurements and reference planes are presented in Figure 5. Pre-treatment and post-treatment PAS measurements and superimpositions are presented at Table 2 and Figure 8, respectively. PM and NOA planes were used for PAS superimpositions.

**Table 2:** PAS analysis of the patient

	**Pre-treatment**	**Post-treatment**
**VAL**	72 mm	75 mm
**TL**	81 mm	74 mm
**S-PAS**	17 mm	22 mm
**N-PAS**	7 mm	20 mm
**I-PAS**	16 mm	25 mm
**H-MP**	19 mm	11 mm

**Figure 9:**
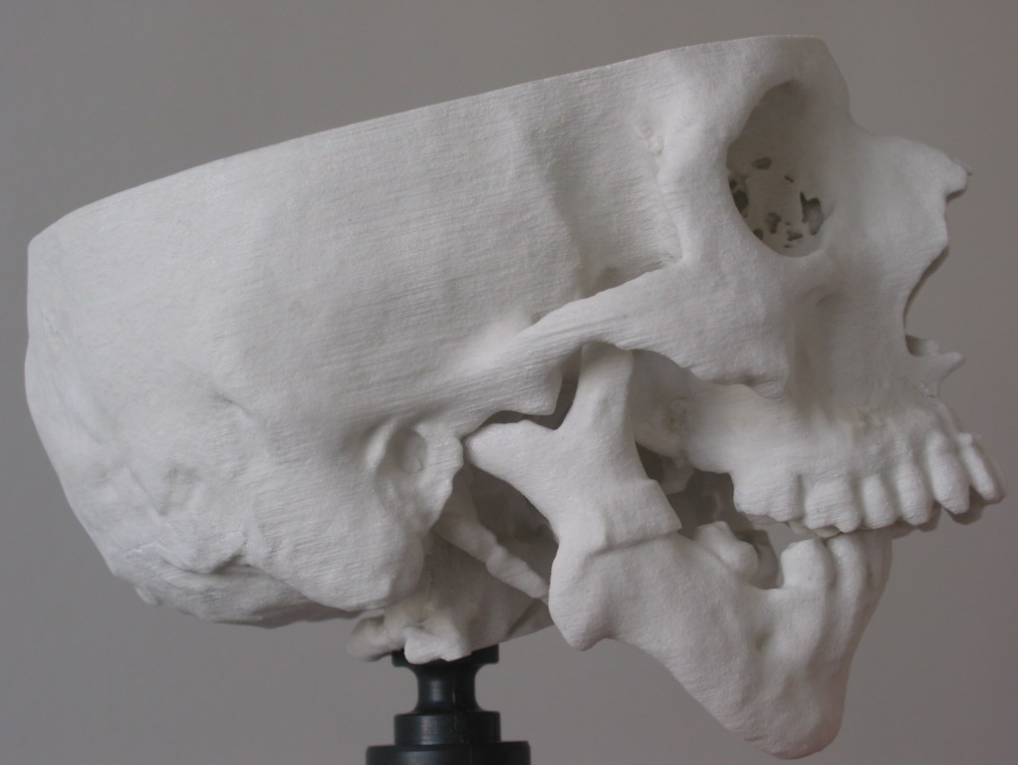
Pre-operative stereolithographic model of the patient.

**Figure 10:**
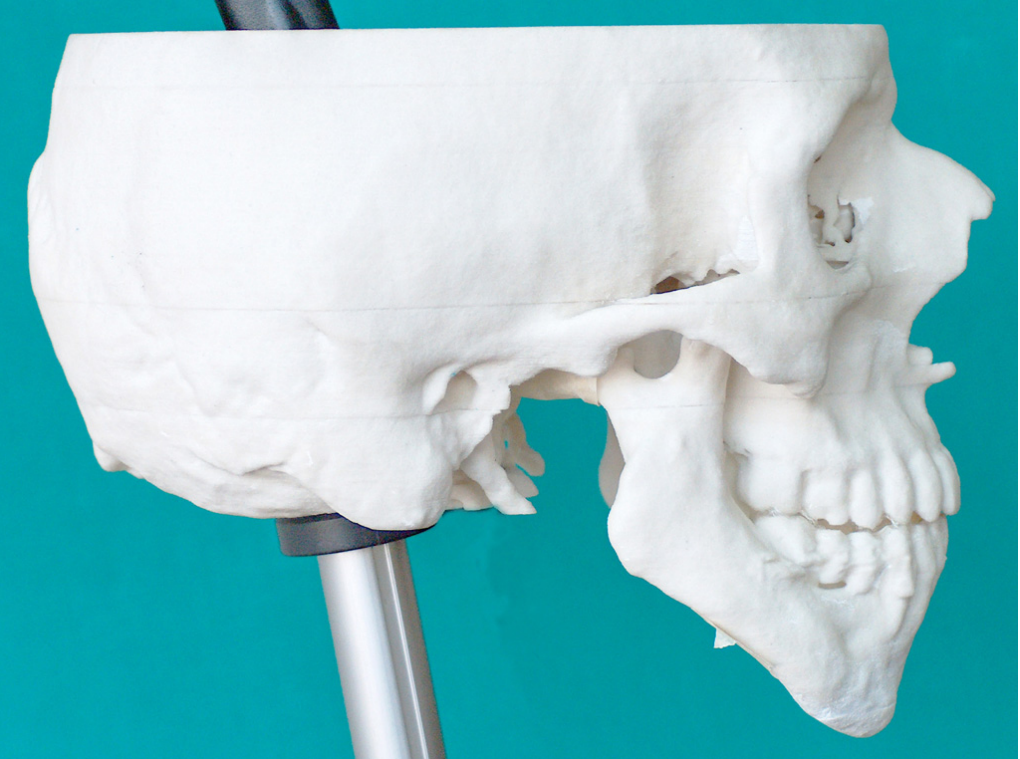
Post-operative stereolithographic model of the patient.

Measurements on the pre- and post-treatment solid models of the patient revealed that ramus lengths increased from 44 mm to 45 mm at right side, from 49 mm to 50 mm at left side while corpus length increased from 69 mm to 80 mm at right side and from 68 mm to 80 mm at left side. Transversal measurements in-between right and left gonion points revealed a decrease from 101 mm to 93 mm. Preoperative and postoperative solid models are shown in Figures 9, and 10.

**Figure 11:**
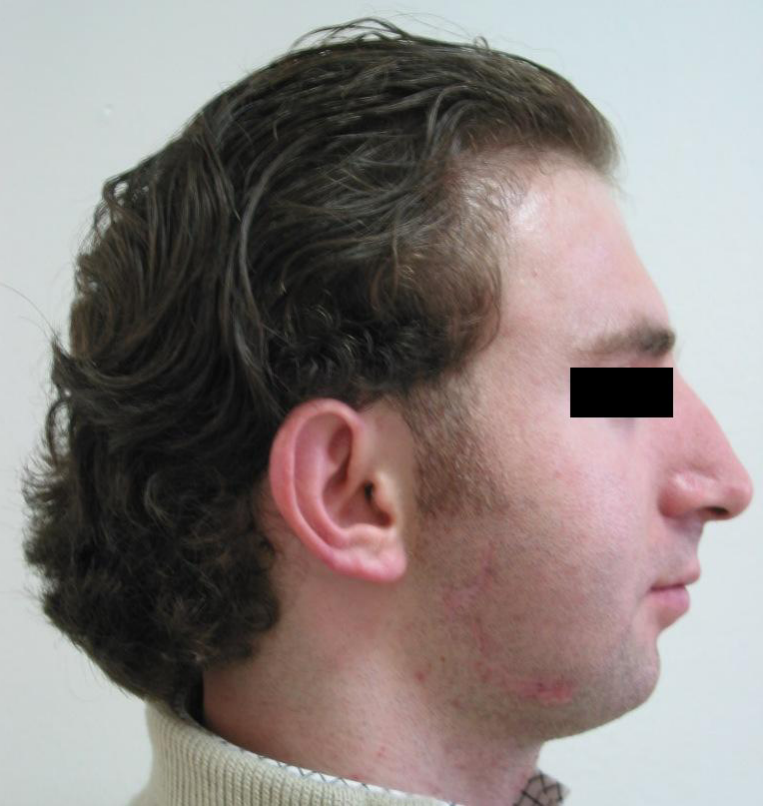
Extraoral lateral view of the patient after treatment.

**Figure 12:**
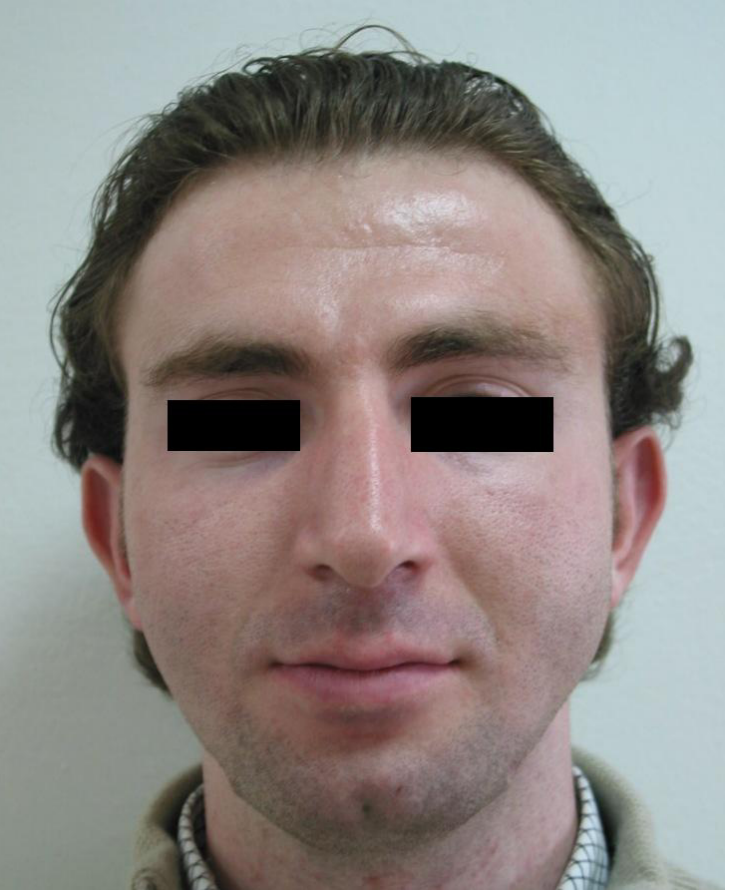
Extraoral front view of the patient after treatment.

**Figure 13:**
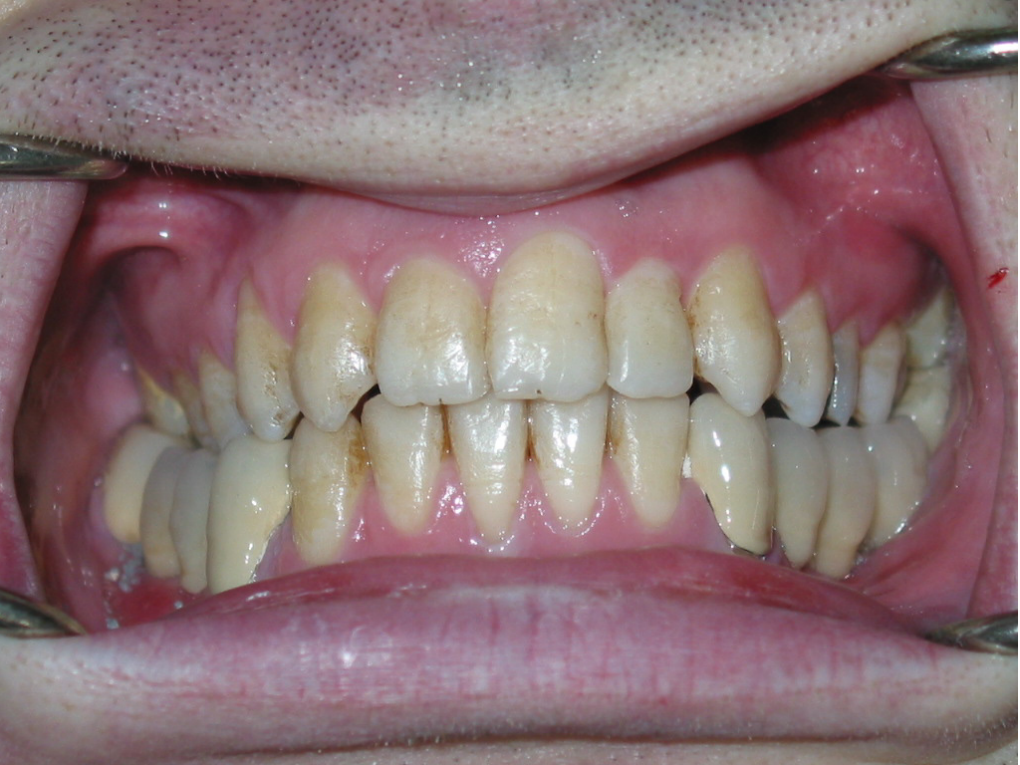
Intraoral view of the patient after treatment.

Satisfactory results from both aesthetic and functional standpoints were acquired via distraction osteogenesis of the ramus and corpus. Correction of the skeletal deformity improved the speech and pronounciation of the patient. Respiratory problems were resolved, wheezing and snoring ended. Although adjunctive aesthetic surgeries such as rhinoplasty, scar revision and angulus augmentation were planned for optimal satisfactory results, the patient was pleasant from his new image and he refused to undergo additional operations. Missing lower teeth were reconstructed with fixed prosthesis 3 months after the treatment. Postoperative extraoral and intraoral photographs are presented in Figures 11, 12, and 13.

## Discussion

Mandibular hypoplasia is one of the most common malformations of the facial skeleton. It is usually associated with a deficient gonial angle, ascending ramus, and mandibular corpus. Maxilla, zygoma, temporal bone, cranial vault and cervical spine are the other anatomic landmarks that may be affected [1,7]. Pruzansky [7] classified mandibular hypoplasia according to the severity of the deformity. In this classification, as the severity increases the ascending ramus is affected to a greater degree.

In the presented case, the patient had a severe mandibular hypoplasia due to short ramus and mandibular body. Since mandibular lengthening procedure required vertical and horizontal components of distraction, an extraoral multiplanar distractor was selected. Intraoral devices usually apply unidirectional force due to the limited space in the oral cavity. Problems such as malocclusion and deficiencies in lower facial contours have been encountered in use of unidirectional devices for mandibular distraction [8]. Extraoral bidirectional or multidirectional distractors should be preferred in the correction of severe deformities including ramus, corpus and the angle of mandible [9,10].

Molina and Ortiz-Monasterio [9] were the first to use bidirectional osteodistraction in the mandible to lengthened both ramus and corpus of the mandible simultaneously. Anatomically, the mandible consists of two halves that are fused at an acute angle in the midline forming V-shaped bone structure. Therefore, in order to correct severe mandibular deformities in three dimensions, independent lengthening of mandibular corpus and ramus must be combined with gradual angular adjustments. Because of this, several multidirectional extraoral distractors have been developed in the last decade [11,12]. Multidirectional distractors are advantageous in distraction of the mandible in all three planes of space [13]. Linear or angular correction in the sagittal and vertical planes and angular correction in the transverse plane can be obtained with a multidirectional distractor and movement of bone segments or shape of the regenerate bone may be changed during distraction process [12]. In our patient, we used a multidirectional distractor and performed two ipsilateral osteotomies to shorten the total distraction period by creating two callus sites. This also allowed the development of a mandibular angle [1,2,12]. In the cephalometric analysis and model measurements we couldn't observe a significant increase in ramus length. Although the vertical component of the distractor was activated for 7 days, it did not reflect to ramus length exactly. Similarly, horizontal component was activated 45 mm but corpus length increased only 18 mm according to the cephalometric analysis. In our opinion the reason of the relapse, which was more than expected, was probably depending on the angular alterations performed during distraction period on days 10, 20, 30 and 40. These angular alterations were made in order to decrease the gonial angle and create anterior rotation of the mandible.

In the treatment of the patient, distraction osteogenesis was preferred as orthognathic surgery has relapse risk in severe mandibular deficiencies requiring lengthening of the mandible more than 8–10 mm. Even when the surgical technique is modified, 15 mm is approximately the outer limit of predictable surgical mandibular advancement. In our patient, lengthening of the ramus was also needed but after conventional orthognathic surgery procedures, pterygoid muscle usually does not adapt to the elongation of ramus. However, during distraction osteogenesis, active histiogenesis occurs in different tissues including gingiva, blood vessels, ligaments, cartilage, muscles and nerves [15,16]. These adaptive changes in the soft tissues decrease the relapse risk and allow the treatment of severe facial deformities.

DO has also some risks such as infection, loosening of the distractor, paraesthesia, and excessive skin damage caused by the pins of the extraoral device. Strategic mistakes such as inappropriate distractor configuration or inadequate calculation of distraction parameters and technical mistakes like misalignment of the distractor leading to displacement of the bone segment, insufficient rate of the lengthening, premature consolidation may cause undesired results. These complications are usually related with the experience of the surgeon [17-19]. In the presented case, only facial scar was observed that was the inevitable result of the extraoral distractor.

Severe mandibular hypoplasia can lead to a reduction of oropharyngeal capacity and glossoptosis because of the posterior location of the insertion of the suprahyoid muscles into the mandible. As a result, upper airway obstruction, feeding difficulties, gastroesophageal reflux may occur. Several authors have reported that these conditions could be resolved by the help of mandibular distraction [20-22]. Similarly, following the advancement of the mandible, respiratory problems, snoring, and difficulties during feeding and talking improved in our patient, due to the increase in the PAS and correction of the excessive overjet.

Elongation of the corpus length increased the volume of the oral cavity therefore tongue was relieved. Before treatment, tongue tip was positioned over the incisal edges of the lower incisors because of insufficient space. However, following the treatment, it retruded behind these teeth to a normal position. This retrusion decreased the value of TL (distance between tongue tip and Eb). The tongue accompanied the advancement of the mandible and postural alterations occurred. Since the tongue moved in the anterior direction, all the PAS parameters increased. The most significant increase was in the N-PAS distance which was threefold higher than the initial value. Translation of the tongue affected not only the width of the N-PAS but also changed the position of this area. N-PAS was under I-PAS before treatment but it positioned between S-PAS and I-PAS following the treatment and this was the place where it should be. During the distraction of the corpus, mandible revealed anterior rotation and probably this rotation affected the hyoid bone through the muscles and caused an elevation at the hyoid so the perpendicular distance from hyoid to mandibular plane decreased after treatment.

During the treatment planning, both cephalometric analysis and solid model measurements were utilized. Solid model revealed the exact shape and size of the skeletal structures and provided the advantage of measuring the length of ramus and corpus directly and separately for right and left sides. Model surgery may be performed on the solid model so as to determine the osteotomy sides, inclination of the osteotomy and distraction vector. Distractors may be placed on the model after the model is cut at the osteotomy side with a saw and it may be activated to observe the results that would occur at the end of treatment and to determine the distraction period. In our opinion solid models could be used successfully in the treatment planning of distraction osteogenesis or orthognathic surgery cases.

## Competing interests

4C Engineering, Materialise and Z Corporation have declared a potential conflict of interest. 4C Engineering-Istanbul, Turkiye is the partner company of Materialize Co- Belgium. Materialise is the developer of Mimics 3D image processing and editting software. 4C engineering uses Spectrum Z510 Color 3D Printers which is produced by Z Corporation-Burlington, USA. All other authors have declared that no competing interests exist.

## Authors' contributions

KO performed the surgery of the patient and contributed to writing the paper. SK did the orthodontic treatment. MS was the assistant surgeon. EA performed the cephalometric analysis, PAS measurements and superimpositions. AHO was the second assistant surgeon and wrote the paper. OB planned the treatment procedures. All authors gave useful comment on the analysis of data and text of the manuscript. They read and approved the final manuscript.
